# The Progress of the Vaccine

**Published:** 1801-06

**Authors:** 


### *The Progreſs of the Vaccine.*

WE learn from the French Official Paper, of the 5th and 8th Ventoſe, that the new Inoculation makes great progreſs in France. After naming the committee appointed to ſuperintend

---

tend the firſt experiments, they ſay, " They have had the ſatisfaction of ſeeing their labours crowned with all the ſucceſs they could deſire for the time. The Vaccine ſpreads with great rapidity among all claſſes of ſociety; and in the departments where the ſmall-pox has produced terrible ravages during the laſt years. The trials made at Vaugirard, in the houſe and under the direction of Citizen Colon, have furniſhed a kind of ſchool, and a ſource of rays which this young phyſician, by his ſingle exertions, has rendered luminous, with a rapidity and ſucceſs which have merited for him the thanks of the public at large.

The number already inoculated amounts to ſeveral thouſands; and a great part of the phyſicians of the departments are haſtening to confer the benefits of this method upon their fellow-citizens. We ſhall, perhaps, be excuſed, if we merely give here the information we have derived from Citizen Colon himſelf, and the authenticity of which he guarantees.

Inoculated by him at Paris, of both ſexes and every age, 250. By others: At Gand, 150; at Arras, 70; at Nancy, 120; at Rouen, 151; at Lille, 100; at Saint-Tulle, near Marſeilles, 147; at Verſailles, by practitioners whoſe names are alſo mentioned, 227; at Toulouſe, 40; at Narbonne, 60; at St. Quentin, and its neighbourhood, 300; at Lombes, 30; at Riom, 80;—Total, 1764.

In this number are not compriſed, thoſe who have been inoculated by thirty-ſix other phyſicians or ſurgeons, to whom he has ſent vaccine virus; nor a great number of inoculations performed with ſucceſs in Paris, by Citizens Guillotin, Thouret, Leroux, Cattet, Portal, and other practititioners, well known and eſteemed by the public.

An important remark to be made on this ſubject is, that the government has exerted no influence whatever on the operations neceſſary to demonſtrate the advantages of the new inoculation; that its intervention has been confined to the facilitating the means of obtaining young children from the hoſpital of Maternity, who have been taken care of and attended at the houſe of Citizen Colon, with all the attention and tenderneſs which they could have experienced in the houſe of their parents. Every thing elſe has been ſo entirely committed to the ſkill, the zeal, and the intelligence of the Medical Committee that no kind of reſtraint has been put on the opinions or the conduct of the phyſicians who have conducted this important experiment. The ſucceſs of it is now eſtabliſhed. It is demonſtrated, that it preſerves from the ſmall-pox; and the laſt trials made at the Prefecture of Paris have, on this point, produced the higheſt degree of conviction.

In thefe trials, which were made on the 11th Nivofe, and which the phyficians called the *Counter-proof*, feven young children who had been *vaccinated* from three to four months before by Cit. Colon, were inoculated with fmall-pox matter by Cit. Ané, the well-known inoculator, in the prefence of many medical practitioners. The variolous matter was taken by Cit. Evral from a child of Cit. Frochot, who then laboured under a copious eruption of the natural fmall-pox. Of thefe feven children, thus inoculated, in three places each, not one took the fmall-pox, which proves, in the moft fatisfactory manner, the efficacy of this method as a prefervative againft it.

Citizen Frochot is not the only magiftrate who has been defirous of determining clearly, and under his own obfervation, the utility of the vaccine; the zeal and care exercifed at Saint Quentin by Cit. Dunes, Sub-Prefect of this diftrict, who, to emancipate his fellow-citizens from the ravages caufed laft year by the fmall-pox, was eager to invite Cit. Colon to St. Quentin, to propagate and naturalize the vaccine, which has been done with the greateft fuccefs.

Moreover, very recently, Cit. Corbigny, Præfect of the department of Loir et Cher, had fent expreffly to Paris Cit. Defperanches, a furgeon of eminence at Blois, accompanied by a young child, to purfue the treatment of the vaccine at the houfe of Cit. Colon, and to carry back the vaccine virus into his department, with a view to propagate and naturalize it.

Such are the facts within our knowledge, and of which we think it our duty to inform the public and our neighbours, who may wifh to profit by our experience.

We will only add one word more upon the application of this difcovery to the adminiftrators of the government.

According to all the records of medicine, the fmall-pox is fatal to one out of ten which it attacks.

The vaccine, which abfolutely guards from the fmall-pox, preferves to the community all thofe who would have been carried off by the fmall-pox.

If the proportion of thefe to thofe dying of every other difeafe were afcertained, we fhould know how many we fave annually by the means of the new inoculation.

It appears from the ftatement of Cit. Mourges, in his Statiftical Effay, that out of 1183 deaths at Montpelier, in the years 1774, 1778, and 1783, on an average of the three years, there died of the fmall-pox 420.

The Tables of Cit. Bottin, inferted in the Annuaire of the Bas Rhin, prove, that of 2170 deaths, 158 were victims of the fmall-pox.

Admitting this proportion, which does not depend on a particular ticular epidemic, as applicable to all the departments, the refult will be, that the number of deaths by the fmall-pox is one in fourteen.

The number of deaths in France, then, amounts, on an average, to 900,000, and of this number the fmall-pox, on an average, carries off 64,285 5-7ths.

The refult of which proves, that there is an indiffoluble connection between the advancement of ufeful fcience and the ftrength and profperity of ftates."

---

